# Ethnic and geographic variations in multiple chronic conditions among community-dwelling older people in Xinjiang: a cross-sectional study

**DOI:** 10.1186/s12877-023-04159-8

**Published:** 2023-07-24

**Authors:** Zhuoya Maimaitiwusiman, Aishanjiang Wumaier, Wenwen Xiao, Saiyare Xuekelati, Buluhan Halan, Hong Xiang, Hongmei Wang

**Affiliations:** The Second Ward of the Health Center for Cadre of People’s Hospital of Xinjiang Uygur Autonomous Region, No. 91, Tianchi Road, Urumqi, Xinjiang China

**Keywords:** Aging, Multiple chronic conditions, Cross-sectional studies

## Abstract

**Background:**

Multiple chronic conditions (MCC) refer to the presence of two or more chronic diseases. The incidence of MCC is higher in older people, and increases with age. Studies have shown an association between MCC and increased adverse outcome, as well as the higher mortality, decline of physical function, and poor quality of life in older populations. Herein, for the first time we provide the data of ethnic and geographic variations in the prevalence of MCC among community-dwelling older people in Xinjiang, China.

**Methods:**

A multilevel random sampling method was employed to perform an epidemiological survey in community-dwelling older adults in southern, northern, and eastern Xinjiang between January 2019 to December 2019. In total, 87,000 participants volunteered, with a response rate of 96.67%; 490 participants with incomplete data were excluded and data from 86,510 participants were analyzed.

**Results:**

Our analysis demonstrated that hypertension (51.5%), obesity (27.0%), diabetes (16.9%), heart disease (8.2%), and anxiety/depression (4.5%) were the five main chronic diseases in Xinjiang. The prevalence of MCC in the population surveyed was 33.4% (95%CI, 33.1–33.7). The prevalence in females was 36.6% (95%CI, 36.1–37), which was higher than that in male (30%,95%CI, 29.5–30.4). The prevalence of MCC in older people aged 60–69, 70–79, 80–89, and ≥ 90 years was 32.7% (95%CI, 32.2–33.3), 34.3% (95%CI, 33.9–34.7), 32.5% (95% CI, 31.7–33.3), and 25.9% (95% CI, 23.5–28.3) respectively. As far as the ethnic group was concerned, the prevalence of MCC in Uygur, Han, Kazak, Hui, and Mongolian was 31.3% (95%CI, 30.9–31.7), 34.4% (95%CI, 33.9–35), 40.4% (95%CI, 39.1–41.8), 40.8% (95%CI, 38.9–42.7), 44.4% (95%CI, 38.1–50.6), respectively. The prevalence of MCC was 32.8% (95%CI, 32.1–33.5), 31.7% (95%CI, 31.2–32.1), 36.0% (95%CI, 35.3–36.7) and 39.2% (95%CI, 38.1–40.3) among uneducated, educated for 1–6, 7–9, and more than 10 years. After adjusting for age, sex, smoking, alcohol consumption, and education by level, the binary logistic analysis showed that, compared with eastern Xinjiang, the risk of MCC in the southern area was increased (odds ratio [OR], 1.418; 95% confidence interval [CI], 1.367–1.471), and it was relatively higher in northern Xinjiang (OR, 2.021; 95% CI, 1.944–2.102). Compared with Uygur, Han, Kazakh, Hui and Mongolian had an increased risk of MCC, which was (OR, 1.075; 95% CI, 1.040–1.111), (OR, 1.414; 95% CI, 1.332–1.501), (OR, 1.515; 95% CI, 1.396–1.644), (OR, 1.566; 95% CI, 1.216–2.017), respectively.

**Conclusions:**

There are ethnic and geographic variations in multiple chronic conditions among community-dwelling older people in Xinjiang. The older adults living in northern and southern Xinjiang and Han, Kazakh, Mongolian and Hui older adults have a higher risk of MCC.

## Background

Multiple chronic conditions (MCC) refer to the presence of two or more chronic diseases [1]. Because of the increase in the aging population, there is a higher prevalence of chronic diseases. Previous data has shown that the incidence of MCC is higher in older adults, and increases with age [2]. Another study demonstrated that the incidence of MCC in low-income individuals was 26.8%, which was two-fold higher than that in high-income individuals (13.4%). Those with 65 to 84 years of age had a 64.9% incidence of MCC [3]. The prevalence of MCC was shown to range from 37.9% in the District of Columbia to 64.4% in West Virginia [4]. According to the global chronic disease report by the World Health Organization (WHO) in 2012, approximately 38 million people worldwide died of chronic noncommunicable diseases, accounting for 68% of total deaths in the world [5]. A Report on the Status and Development Trends of Chronic Diseases in the Older Adults in China showed that the prevalence of chronic diseases in individuals aged 60 years or older was 54.0%, the average number of hospitalization days was 27.43 per year, while the average total hospitalization cost was 2618.16 CNY [6]. On the other hand, a Report on the Status of Nutrition and Chronic Diseases of Chinese Residents (2015) indicated that the death rate due to chronic noncommunicable diseases in China accounts for approximately 86.6% of the total deaths [7]. The prevalence of MCC is high and is related to mortality, decline of physical function, and poor quality of life in older adults [8, 9]. Furthermore, MCC is associated with a low cure rate, high incidence of complications, and high disability as well as fatality rates [9, 10]. Besides, patients with MCC require long-term care with high treatment costs that impact their family and national economic development [11, 12]. Therefore, MCC is a major public health concern that threatens the current and future healthcare systems in many countries around the world.

The significant negative impact caused by MCC requires early prevention, detection, and treatment. Epidemiological survey data help formulation of effective disease prevention and control measures. Located in northwest China and the hinterland of Eurasia, Xinjiang has been a multi-ethnic region since ancient times. According to the Announcement of the Seventh National Population Census released in 2021, there are 2.917 million people aged 60 or above in Xinjiang, accounting for 11.28 per cent of the total population [13, 14]. By consulting the “CHINA STATISTICAL YEARBOOK”, “Outline of the Healthy China 2030 Plan” and “Outline of the Healthy Xinjiang 2030 Plan”, the life expectancy of the whole country is 79 years old in 2030, while the average life expectancy of the Xinjiang population is 76.35 years old [15]. The number of population of older people in Altai, Tacheng, Hami, Turfan, Hetian and Kashgar of Xinjiang is 75,768, 169,630, 99,270, 72,234, 179,082, 382,072 respectively [13]. However, the data of epidemiological survey on MCC for community-dwelling older adults in Xinjiang are largely lacking. Here, we conducted an epidemiological survey in community-dwelling older adults in different geographic regions of Xinjiang to understand the prevalence and risk factors of chronic diseases and MCC in Xinjiang.

## Methods

### Study population

This study was reviewed and approved by the Ethics Committee of the People’s Hospital of Xinjiang Uygur Autonomous Region. All participants provided a written informed consent.

**Fig. 1:**
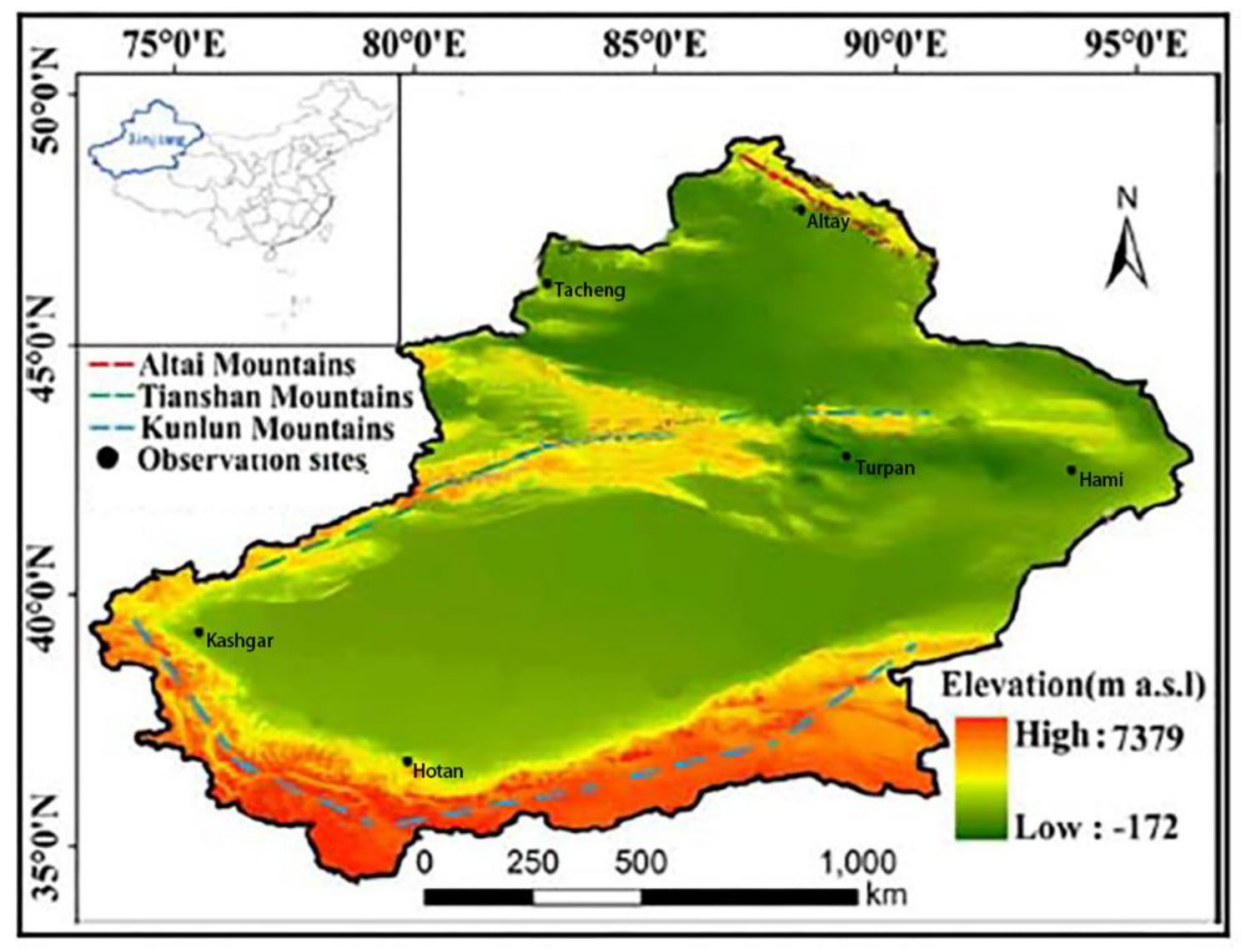
Observation sites in the study, southern Xinjiang, northern Xinjiang, and eastern Xinjiang, China

**Fig. 2:**
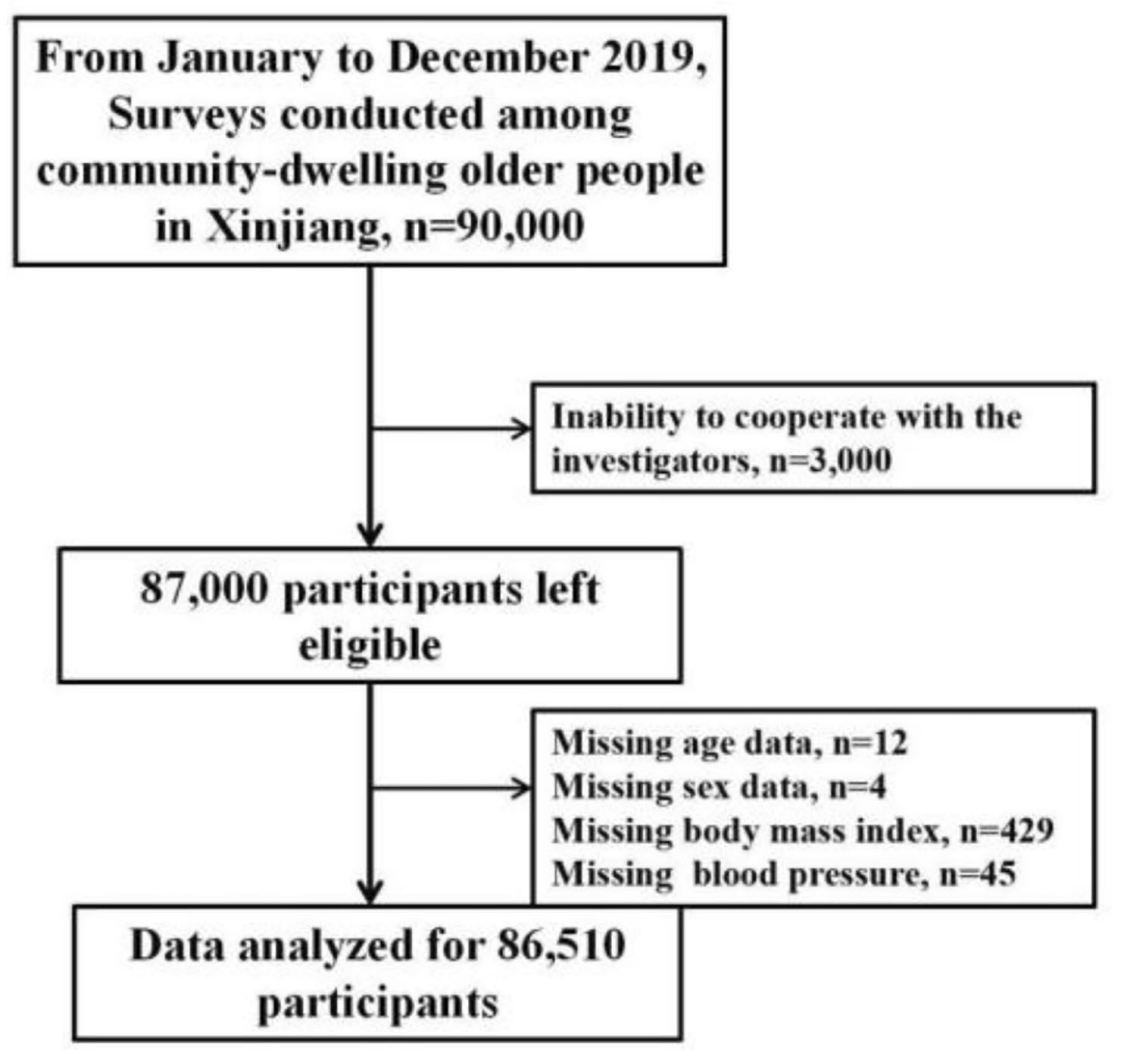
Selection procedure of the study participants

Epidemiological survey and recruitment were performed as following: A multilevel random sampling method was used to perform the surveys of MCC on older adults in Xinjiang from January 2019 to December 2019 [16]. In the first stage, two regions were randomly selected from the southern Xinjiang (Hotan and Kashgar), northern Xinjiang (Altai and Tacheng) and eastern Xinjiang (Hami and Turpan) regions (Fig. 1). In the second stage, one city was randomly selected from each region. The third stage involved two randomly selected counties from each city. During the fourth stage, community-dwelling older adults aged 60 years or older were randomly selected from each county. A total of 90,000 participants were selected for this study. A total of 87,000 individuals participated in the survey, and the response rate was 96.67%. We excluded 490 patients who provided incomplete data. Therefore, we used data from a total of 86,510 patients for statistical analyses (Fig. 2).

The study included participants who were 60 years or older, those who were able to complete the survey, understand the investigation, cooperate with investigators as well as those with the ability to provide informed consent. The criterion of exclusion was the inability to cooperate with the investigators.

### Questionnaire

Every participant completed the standard questionnaire as previously described [17] with the guidance of a physician. The standardized questionnaire was developed by the coordinating center, Fuwai Hospital, CAMS & PUMC (Beijing, China). In brief, data on demographic and other factors, including education, occupation, and lifestyle (e.g. smoking, alcohol consumption, drink tea, diet, physical activity status, etc.) were recorded in interview. For each participant, self-reported history of CVD, which was defined as coronary heart disease and stroke, was collected and verified with medical or hospital records.

### Physical examination

Each participant underwent a detailed physical examination by a physician. Blood pressure, weight, height, and waist circumference were measured according to standard methods.

Systolic and diastolic blood pressure measurements were obtained following the guidelines by the American Heart Association. All participants involved in this study were prohibited from smoking and drinking alcohol, tea, and coffee for 30 min before the blood pressure measurements, which were obtained three consecutive times (10-minute rest between measurements) on the right upper arm using a desktop mercury sphygmomanometer while the participant was in a sitting position. The room temperature was kept at more than 18 °C when measuring blood pressure.

We measured weight after fasting using a calibrated medical scale on which the participants stood upright. The participants were asked to remove their shoes and only wear light clothes during weight measurements. The scale measurement was accurate to within 0.1 kg.

Height measurements were performed using a ruler with a minimum scale of 1 mm that was fixed vertically to the ground. The participants stood upright, with both heels close to the measuring ruler; while the shoulders and hips were close to the measuring ruler. The surveyor placed a square ruler on top of the participant’s head. Two sides of the right angle were close to the measuring ruler, while the other side was close to the participant’s scalp. The measurement was accurate to within 1 mm.

The waists were measured while the participant was upright with the feet 30 to 40 cm apart. An inelastic soft ruler with a minimum scale of 1 mm was placed on the right mid-axillary line, at the midpoint of the line between the upper edge of the hip and the lower edge of the twelfth rib (the natural narrowest part of the waist). The measurement was accurate to within 1 mm. The body mass index (BMI) was calculated as weight (kg)/height (m^2^).

### Blood sample collection and laboratory analyses

Disposable blood collection equipment was used to draw 10 mL of peripheral venous blood in the morning from the participants who had fasted for at least 10 h. The samples were centrifuged immediately to separate plasma (serum) from blood cells, which were then stored at -80℃. At the Xinjiang Uygur Autonomous Region People’s Hospital (third-level A hospital), Hitachi 7600 biochemical analysis system and biochemical indicators such as nitrogen were used for analysis of fasting blood glucose, total cholesterol, triglycerides, high-density lipoprotein cholesterol, low-density lipoprotein cholesterol, serum creatinine, and serum urea.

### Diagnostic criteria

Hypertension was diagnosed as systolic blood pressure ≥ 140 mmHg and/or diastolic blood pressure ≥ 90 mmHg. The participants were also considered to have hypertension if they had been diagnosed with and treated for hypertension previously. Diabetes was diagnosed as fasting plasma glucose ≥ 7.0 mmol/L. The participants were also considered to have diabetes if they had been diagnosed with and treated for type 2 diabetes previously. Abdominal obesity was diagnosed following the Guidelines for the Prevention and Control of Overweight and Obesity in Adults in China. Men with a waist circumference ≥ 85 cm and women with a waist circumference ≥ 80 cm were considered to have abdominal obesity. We defined participants as smokers and consumers of alcohol following the standard guidelines by the World Health Organization (WHO): continuous smoking or smoking for ≥ 6 months and consumption of at least 8 g of alcohol ones per week.

Diagnoses of other diseases were obtained through self-reports by the participants during the investigation, the participants were asked whether a physician ever told them they had chronic diseases such as heart disease (angina pectoris, myocardial infarction, stent/coronary bypass, heart failure, arrhythmia), anxiety/depression, cognitive impairment, hearing impairment, eye diseases (cataract, papilledema, retinal hemorrhage or exudation), cerebrovascular disease (transient ischemic attack, cerebral infarction, cerebral hemorrhage), chronic lung disease (chronic bronchitis, chronic obstructive pulmonary disease, tuberculosis, asthma, emphysema, bronchiectasis, pneumoconiosis), chronic kidney disease (chronic nephritis, nephrotic syndrome, renal insufficiency, kidney stones, polycystic kidney disease) or osteoarthritis/rheumatoid arthritis.

### Quality control

This study used standard methods and standardized instruments to collect relevant data. To control potential errors from observers, the investigators and researchers performed questionnaire survey, physical examination, blood sample collection, transportation, separation, preservation, marking, recording, questionnaire review, and data input. According to the principle of double-blind data input, two professionals loaded the data in parallel and a statistical analysis was performed for verification.

### Statistical analysis

SPSS 23.0 (SPSS Inc., Chicago, Illinois, USA) statistical software was used for data processing. Continuous variables with Gaussian distribution were presented as mean (standard deviation); non-gaussian distribution variables were reported as median (interquartile range); while count variables were expressed as number (percentage). For comparing the differences of clinical phenotypic measurement variables between the two sexes, the student’s t-test or Mann-Whitney U test was applied for Gaussian or non-gaussian distribution variables; while the chi-square test (χ2) was used to compare count variables. At the same time, descriptive analysis was carried out on the distribution of chronic diseases in older people of different genders, ages and geographic in the Xinjiang community, which was expressed as a percentage. We also calculated the prevalence (%), 95% confidence intervals (95% CI), and P values of MCC for the overall population, older adults by gender, age, geographic, ethnic group, and education level, using the Pearson chi-square test. After adjusting for confounding factors (age, sex, smoking, alcohol consumption and education level), multivariate logistic regression was used to analyze the influence of ethnic and geographic variations on MCC.

## Results

### The clinical characteristics of the selected participants

Analyses of the clinical characteristics of the selected participants showed that women had statistically significantly higher BMI, heart rate, fasting blood glucose, total cholesterol, triglycerides, low-density lipoprotein cholesterol, and high-density lipoprotein cholesterol compared to men (*P* < 0.001) (Table 1). The levels of serum creatinine, urea nitrogen, alanine aminotransferase and aspartate aminotransferase in men were significantly higher than in women (*P* < 0.001). In addition, there were fewer women who smoked and consumed alcohol (*P* < 0.001). Besides, 24.7% of women lacked education compared to 16.9% in men.

**Table 1:** Characteristics of study participants in Xinjiang, 2019. (n = 86,510)

	Male(n = 41,188)	Female(n = 45,322)	*T/Z/χ²*	*P*
Age, year, mean (SD)	73.59(6.10)	73.33(5.94)	6.382	< 0.001
BMI, kg/m², mean (SD)	25.26(3.99)	26.12(4.71)	-28.698	< 0.001
h, bpm, mean (SD)	75.05(12.47)	78.41(11.97)	-39.684	< 0.001
FBG, mmol/L, mean (SD)	5.88(2.08)	6.03(2.21)	-10.569	< 0.001
Scr, µmol/L, mean(SD)	80.64(24.44)	67.39(23.09)	80.133	< 0.001
BUN, mmol/L, mean (SD)	5.88(2.47)	5.51(2.60)	21.219	< 0.001
TC, mmol/L, mean (SD)	4.54(1.27)	4.92(1.31)	-42.427	< 0.001
TG, mmol/L, mean (SD)	1.38(0.94)	1.58(1.02)	-29.694	< 0.001
LDL-c, mmol/L, mean (SD)	2.54(0.92)	2.71(1.03)	-21.355	< 0.001
HDL-c, mmol/L, mean (SD)	1.33(0.78)	1.46(0.86)	-19.702	< 0.001
ALT, U/L, median (IQR)	19.20(14.50-25.75)	17.54(13.39–23.60)	-27.514	< 0.001
AST, U/L, median (IQR)	21.38(17.80–26.00)	21.00(17.10-25.45)	-11.208	< 0.001
Smoking, n (%)			8636.335	< 0.001
NO	33,542(81.4)	45,136(99.6)		
YES	7646 (18.6)	186 (0.4)		
Drinking, n (%)			4084.542	< 0.001
NO	36,958(89.7)	45,048(99.4)		
YES	4230 (10.3)	274 (0.6)		
Education, years, n (%)			1363.214	< 0.001
Uneducated	6958(16.9)	11,198 (24.7)		
1–6	20,314(49.3)	23,194 (51.1)		
7–9	9554(23.2)	7778 (17.2)		
10 years and above	4362 (10.6)	3152 (7.0)		

**Abbreviations**: HR: heart rate; FBG: fasting blood glucose; Scr: Serum creatinine; BUN: Blood urea nitrogen; TC: serum total cholesterol; TG: triglyceride; LDL-c: low-density lipoprotein cholesterol; HDL-c: high-density lipoprotein cholesterol; ALT: alanine aminotransferase; AST: aspartate aminotransferase; SD: standard deviation; IQR: interquartile range

### Distribution of chronic diseases in older adults in Xinjiang

**Fig. 3:**
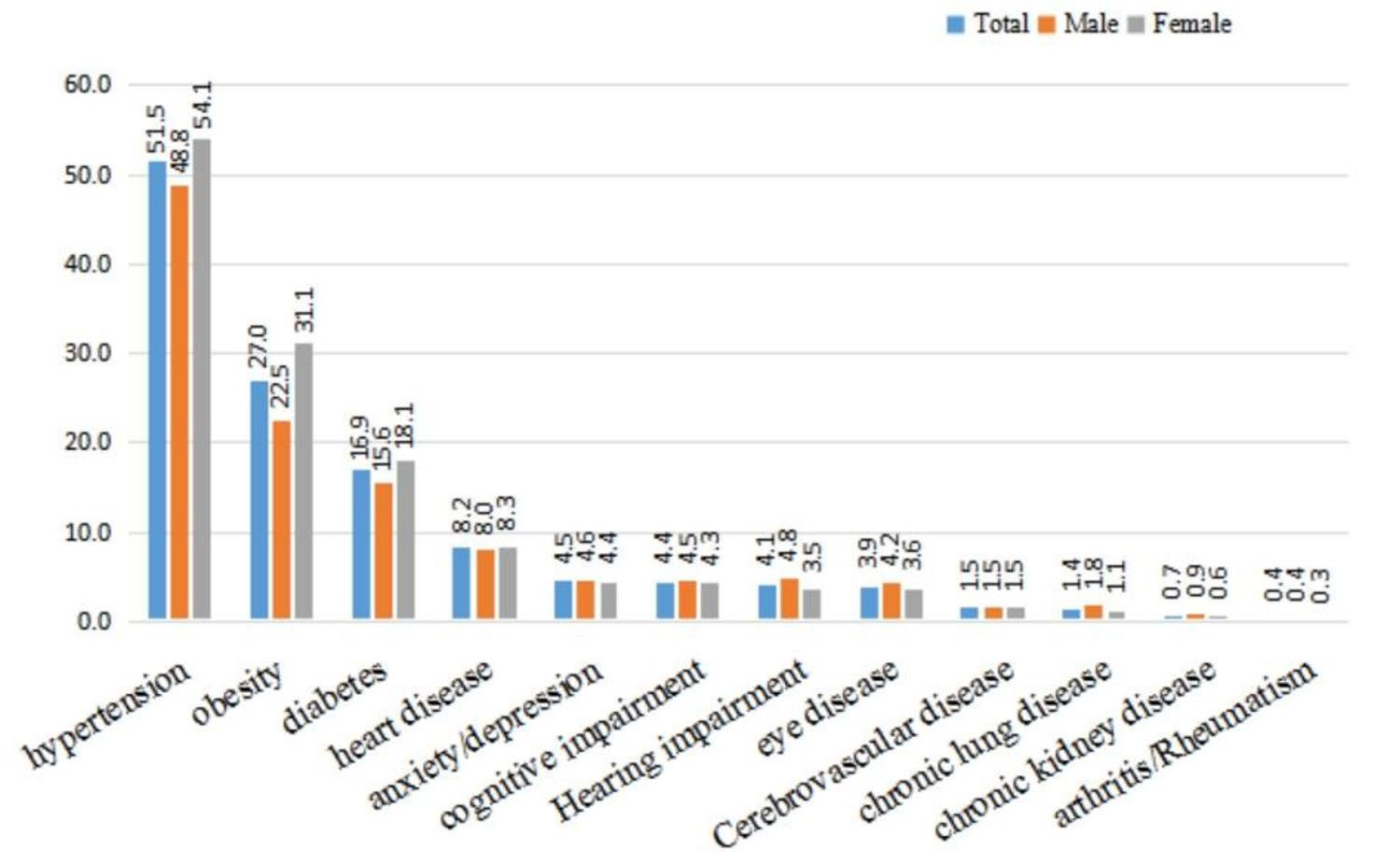
Distribution (%) of chronic diseases among community-dwelling older people (n = 86,510) of different genders in Xinjiang, 2019

Our analysis showed that there were five main chronic diseases among the community-dwelling older adults in Xinjiang, including hypertension (51.5%), obesity (27.0%), diabetes (16.9%), heart disease (8.2%), and anxiety/depression (4.5%), followed by cognitive impairment (4.4%), hearing impairment (4.1%), eye disease (3.9%), cerebrovascular disease (1.5%), chronic lung disease (1.4%), chronic kidney disease (0.7%), and joint inflammation/rheumatoid arthritis (0.4%) (Fig. 3).

**Fig. 4:**
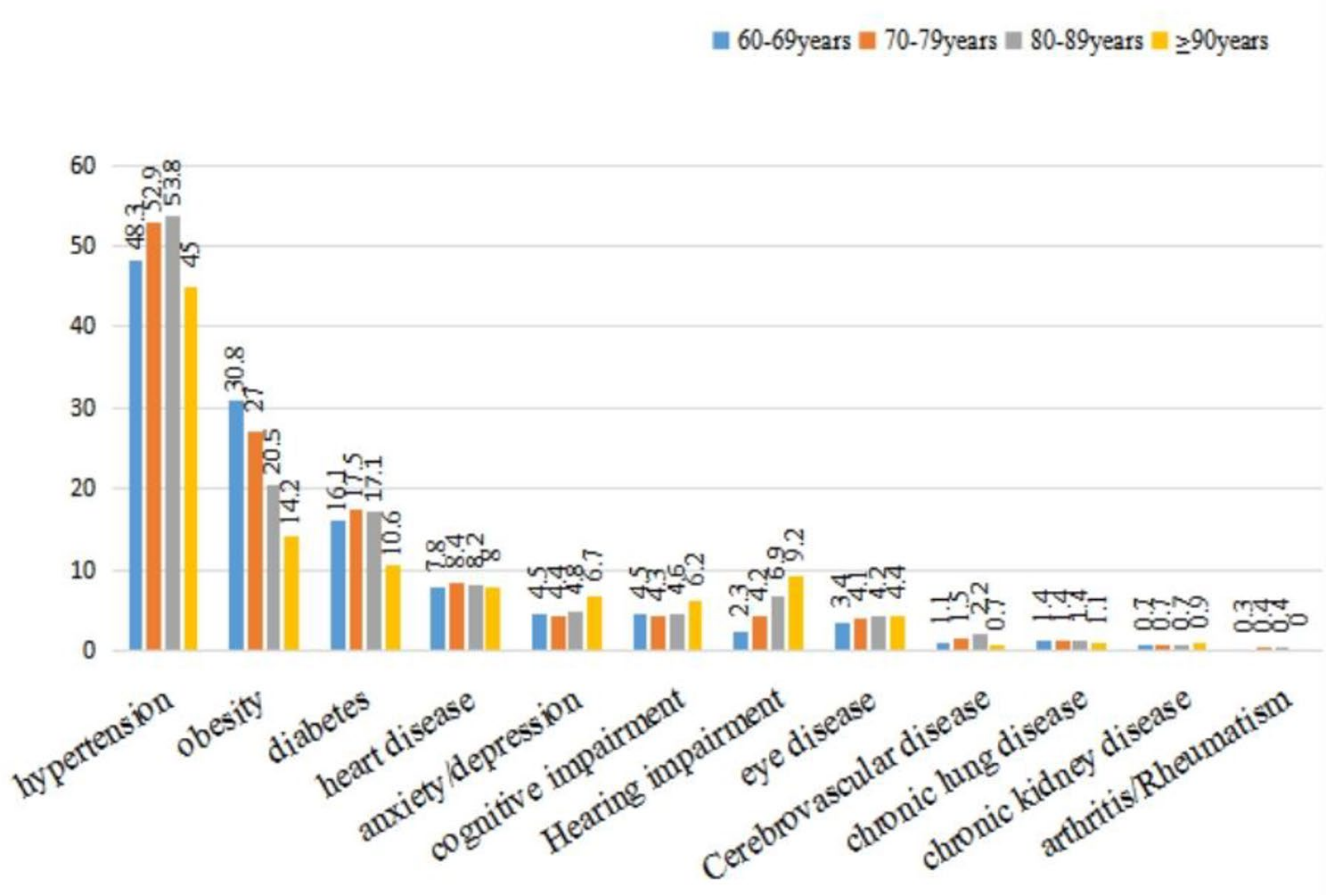
Distribution (%) of chronic diseases in different age groups of community-dwelling older people (n = 86,510) in Xinjiang, 2019

The rates of diabetes and heart disease were the highest in those aged 70 to 79 years (17.5% and 8.4%, respectively), and the rates of hypertension were also higher (52.9%). The rate of obesity was the highest in those with 60 to 69 years (30.8%). The rates of anxiety/depression (6.7%), cognitive impairment (6.2%), hearing impairment (9.2%), cataract and other eye diseases (4.4%) were higher in those aged 90 years or older. The rate of hypertension and cerebrovascular disease in those aged 80 to 89 years were the highest (53.8% and 2.2%, respectively), while the rate of chronic lung disease, chronic kidney disease, and arthritis/rheumatism were not significantly different among the different age groups (Fig. 4).

**Fig. 5:**
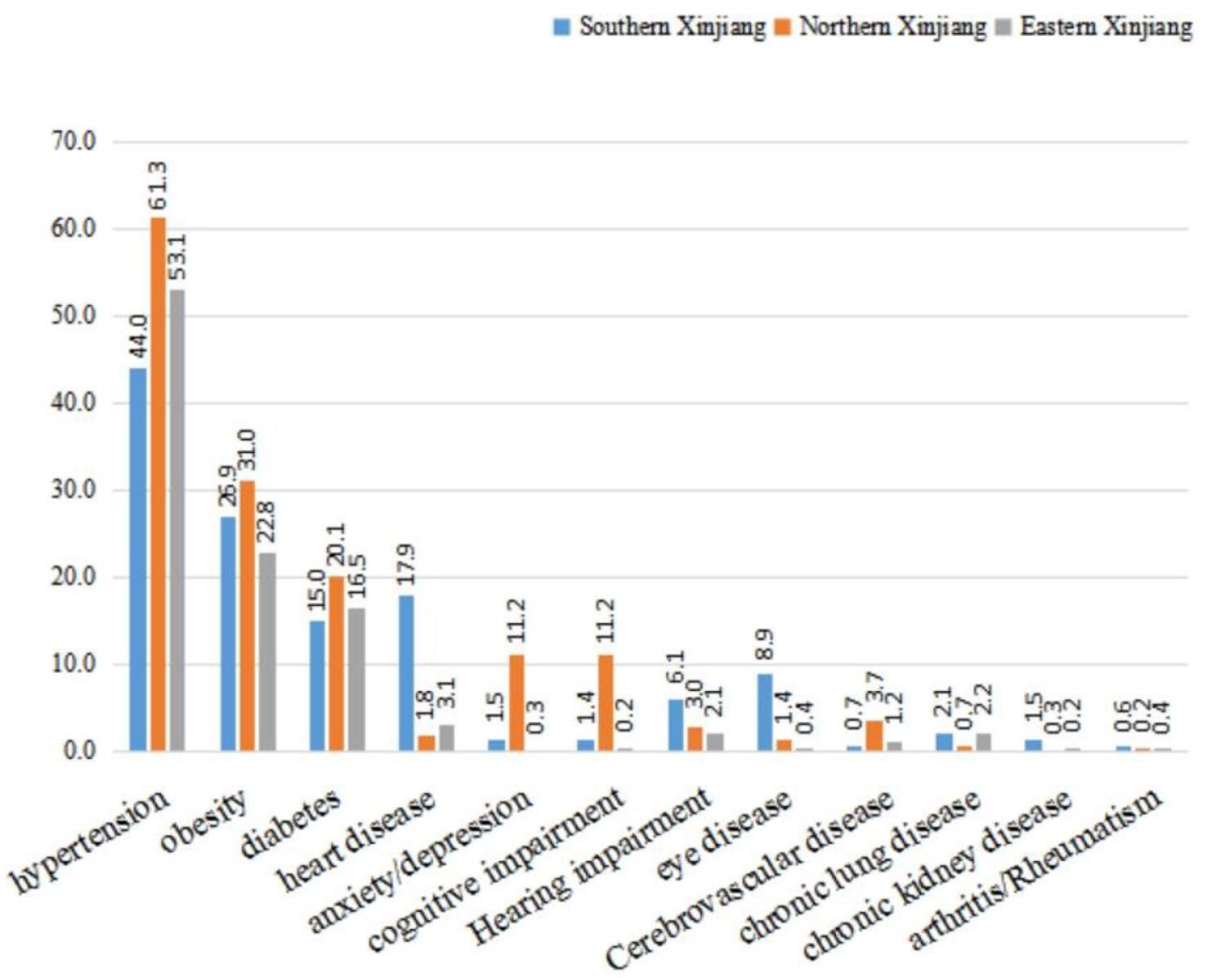
Distribution (%) of chronic diseases in community-dwelling older people (n = 86,510) of different geographic in Xinjiang, 2019

Participants in northern Xinjiang had higher rates of hypertension (61.3%), obesity (31%), diabetes (20.1%), anxiety/depression (11.2%), cognitive impairment (11.2%), and cerebrovascular disease (3.7%) compared to those in southern and eastern Xinjiang. In contrast, the rates of heart disease (17.9%), hearing impairment (6.1%), eye disease (8.9%), chronic kidney disease (1.5%), and rheumatoid arthritis (0.6%) were higher in southern Xinjiang compared to those in northern and eastern Xinjiang (Fig. 5).

### MCC among community-dwelling older adults in Xinjiang

**Fig. 6:**
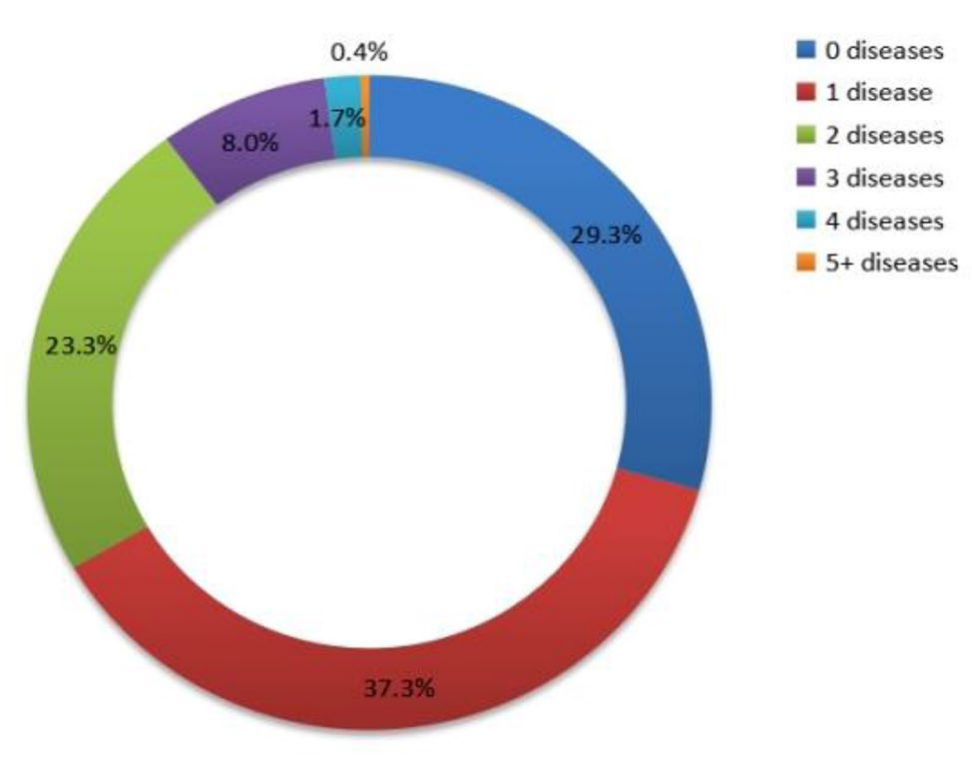
Distribution (%) of the number of chronic diseases in community-dwelling older people (n = 86,510) of Xinjiang, 2019

The prevalence rates of one to five or more chronic diseases were 37.3%, 23.3%, 8.0%, 1.7%, and 0.4%, respectively, and 29.3% of participants had no chronic diseases (Fig. 6). As shown in Table 2, the prevalence of MCC was (33.4%, 95CI, 33.1–33.7) among all participants. However, the prevalence of MCC was higher in women (36.6%, 95%CI, 36.1–37) than in men (30.0%, 95%CI, 29.5–30.4) (*P* < 0.001). The prevalence of MCC was the highest in those aged 70 to 79 years (34.3%, 95%CI, 33.9–34.7) (*P* < 0.001). The prevalence in older people aged 60 to 69 years and 80 to 89 years was 32.7% (95%CI, 32.2–33.3) and 32.5% (95%CI, 31.7–33.3), respectively. The prevalence was lower in those aged ≥ 90 years 25.9% (95% CI, 23.5–28.3). In addition, the rate of MCC in northern Xinjiang (41.7%, 95%CI, 41.1–42.3) was significantly higher than those in southern Xinjiang (32.7%, 95%CI, 32.2–33.2) and eastern Xinjiang (25.8%, 95%CI, 25.2–26.3). Furthermore, the rates of MCC were significantly higher for the Mongolian (44.4%, 95%CI, 38.1–50.6), Kazakh (40.4%, 95%CI, 39.1–41.8), and Hui (40.8%, 95%CI, 38.9–42.7) compared to the Han (34.4%, 95%CI, 33.9–35.0) and Uygur (31.3%, 95%CI, 30.9–31.7) (*P* < 0.001). The highest prevalence of MCC, 39.2% (95% CI, 38.1–40.3), was observed among those who had been educated for more than 10 years (*P* < 0.001) (Table 2).

**Table 2:** Prevalence of multiple chronic conditions among community-dwelling older people of different genders, ages, geographic, ethnic and education level in Xinjiang, 2019. (n = 86,510)

		Total	MCC, % (95% CI)	*χ²*	*P*
All participants		86,510	33.4 (33.1–33.7)		
Genders	Male	41,188	30.0(29.5–30.4)	418.095	< 0.001
	Female	45,322	36.6(36.1–37)		
Age group(years)	60–69	26,424	32.7(32.2–33.3)	59.588	< 0.001
	70–79	45,531	34.3(33.9–34.7)		
	80–89	13,264	32.5(31.7–33.3)		
	≥ 90	1291	25.9(23.5–28.3)		
Areas	Southern Xinjiang	37,856	32.7(32.2–33.2)	1405.202	< 0.001
	Northern Xinjiang	25,130	41.7(41.1–42.3)		
	Eastern Xinjiang	23,524	25.8(25.2–26.3)		
Ethnic	Uygur	48,478	31.3(30.9–31.7)	379.756	< 0.001
	Han	28,237	34.4(33.9–35.0)		
	Kazakh	5138	40.4(39.1–41.8)		
	Hui	2551	40.8(38.9–42.7)		
	Mongolian	248	44.4(38.1–50.6)		
	Others	1858	43.2(40.9–45.4)		
Education(years)	Uneducated	18,156	32.8(32.1–33.5)	230.258	< 0.001
	1–6	43,508	31.7(31.2–32.1)		
	7–9	17,332	36.0(35.3–36.7)		
	10 years and above	7514	39.2(38.1–40.3)		

**Abbreviations**: MCC: multiple chronic conditions

### Geographic and age variations in the prevalence of MCC among the community-dwelling older people in Xinjiang

After adjusting for age, sex, smoking, alcohol consumption, and education by level, the binary logistic analysis showed that compared with eastern Xinjiang, the risk of MCC in the south was increased (odds ratio [OR], 1.418; 95% confidence interval [CI], 1.367–1.471), and it was higher in northern Xinjiang (OR, 2.021; 95% CI, 1.944–2.102). (Table 3). Compared with Uygur, Han, Kazakh, Hui and Mongolian had an increased risk of MCC, which was (OR, 1.075; 95% CI, 1.040–1.111), (OR, 1.414; 95% CI, 1.332–1.501), (OR, 1.515; 95% CI, 1.396–1.644), (OR, 1.566; 95% CI, 1.216–2.017), respectively. (Table 4).

**Table 3:** Unadjusted and adjusted odds ratios (95% confidence intervals) of MCC associated with geographic

	*OR*	*95%CI*	*P*	Adjusted variables
**Model 0**				Unadjusted
Eastern Xinjiang	ref			
Southern Xinjiang	1.398	(1.349–1.450)	< 0.001	
Northern Xinjiang	2.062	(1.984–2.142)	< 0.001	
**Model 1**				Plus gender and age group
Eastern Xinjiang	ref			
Southern Xinjiang	1.420	(1.369–1.473)	< 0.001	
Northern Xinjiang	2.085	(2.006–2.168)	< 0.001	
**Model 2**				Plus gender, age group and education level
Eastern Xinjiang	ref			
Southern Xinjiang	1.412	(1.361–1.464)	< 0.001	
Northern Xinjiang	2.030	(1.952–2.111)	< 0.001	
**Model 3**				Plus gender, age group, education level, smoking and alcohol consumption
Eastern Xinjiang	ref			
Southern Xinjiang	1.418	(1.367–1.471)	< 0.001	
Northern Xinjiang	2.021	(1.944–2.102)	< 0.001	

**Abbreviations**: MCC: multiple chronic conditions

**Table 4:** Unadjusted and adjusted odds ratios (95% confidence intervals) of MCC associated with ethnic

	*OR*	*95%CI*	*P*	Adjusted variables
**Model 0**				
Uygur	ref			Unadjusted
Han	1.153	(1.117–1.189)	< 0.001	
Kazakh	1.491	(1.406–1.582)	< 0.001	
Hui	1.511	(1.393–1.639)	< 0.001	
Mongolian	1.750	(1.361–2.250)	< 0.001	
Others	1.668	(1.518–1.831)	< 0.001	
**Model 1**				Plus gender and age group
Uygur	ref			
Han	1.139	(1.103–1.175)	< 0.001	
Kazakh	1.472	(1.387–1.561)	< 0.001	
Hui	1.515	(1.396–1.644)	< 0.001	
Mongolian	1.693	(1.316–2.178)	< 0.001	
Others	1.661	(1.512–1.825)	< 0.001	
**Model 2**				Plus gender, age group and education level
Uygur	ref			
Han	1.093	(1.059–1.129)	< 0.001	
Kazakh	1.420	(1.338–1.507)	< 0.001	
Hui	1.516	(1.397–1.645)	0.005	
Mongolian	1.570	(1.219–2.021)	< 0.001	
Others	1.608	(1.463–1.767)	< 0.001	
**Model 3**				Plus gender, age group, education level, smoking and alcohol consumption
Uygur	ref			
Han	1.075	(1.040–1.111)	< 0.001	
Kazakh	1.414	(1.332–1.501)	< 0.001	
Hui	1.515	(1.396–1.644)	< 0.001	
Mongolian	1.566	(1.216–2.017)	0.001	
Others	1.592	(1.449–1.750)	< 0.001	

**Abbreviations**: MCC: multiple chronic conditions

## Discussion

The results of this survey demonstrated that the prevalence of MCC in individuals aged 60 years or older was 33.4%, which was consistent with data previously reported in China [18, 19]. Fortin et al. [20] showed results that evaluating 12 or more chronic diseases yield more reliable results on the MCC prevalence. This study analyzed a total of 12 chronic diseases and thus our results were convincing and reliable as well. However, the prevalence of MCC in this study was lower than the findings reported in other countries [4, 21]. Another study in England showed that the prevalence of MCC for middle-aged and older adults was 19%, which was relatively lower than our result described in this study, the reason for that might be that the participants in that study were relatively young [22]. The difference in prevalence may have occurred because the self-reported prevalence of chronic diseases in the older adults was lower than the actual prevalence [23]. Furthermore, there are differences in medical resources and health service levels in different geographic regions [24]. In addition, since the older adults in Xinjiang have insufficient health awareness, the rates of detection of chronic diseases and MCC could be low.

This study showed that the rates of hypertension, obesity, diabetes, and heart disease were higher in women than in men, consistently, the prevalence of MCC was higher in women (36.6%, 95%CI, 36.1–37) than in men (30.0%, 95%CI, 29.5–30.4). These results are consistent with the results of the Fifth National Health Service Survey Analysis Report in China in 2016, which indicated that the rate of chronic disease in women and men were 35% and 31%, respectively [25]. Besides, our findings were consistent with the results of MCC prevalence in each state in the United States in 2017 [4]. The difference in the prevalence rates of MCC for men and women may be due to the different cardiac structures and functions, and psychosocial characteristics [26]. Therefore, there is need for provision of better education regarding prevention and treatment of key chronic diseases and standardization of treatment. Furthermore, the sex difference in the prevalence of chronic diseases to provide a basis for formulating sex-specific MCC prevention and treatment strategies should be focused in the future.

The results of this study showed that the prevalence of MCC in 60–69 years was 32.7% (95%CI, 32.2–33.3), and the highest one was 34.3% (95%CI, 33.9–34.7) in 70–79 years. The prevalence of MCC decreased with age, and the prevalence of MCC was 25.9% (95%CI, 23.5–28.3) at the age of 90 years or older. One reason may be that genetic factors determine the good health status of these long-lived people [27, 28]. In addition, the existing surviving people over 90 years old have a healthier lifestyle [29]. It is also possible that older adults with MCC are more likely to die before the age of 90. This also explains a lower prevalence in the individuals aged 90 years or older. This may also produce a survival bias in this study.

In addition, a cross-sectional study performed in Australia and Japan [30] showed that the MCC rates of the Australian population were 46.0%, 36.1%, and 28.9% for participants with lowest, middle, and highest education level, respectively, while those of the Japanese population were 33.9.%, 24.6%, and 16.6%, respectively. In agreement, a study in Rio de Janeiro showed that the prevalence of MCC for those with primary education was more than twice that of those with a postgraduate education (OR, 2.77; 95% CI, 1.61–4.91) [31]. However, our results led us to the following conclusions, MCC prevalence was 32.8% (95% CI, 32.1–33.5), 31.7% (95% CI, 31.2–32.1), 36.0% (95% CI, 35.3–36.7), and 39.2% (95% CI, 38.1–40.3) among the populations uneducated, educated 1–6 years, 7–9 years, and more than 10 years. The reason may be related to the low proportion of older people with more than 10 years of education. Mass compulsory education had not been implemented before the founding of the People’s Republic of China. However, most of the older people involved in this study were born before the founding of China, resulting in a relatively higher proportion of uneducated group and a lower proportion group with education in high school and college [32, 33]. Therefore, the high prevalence of MCC in well-educated older people in this study may be related to a special historical scenairo in China. Therefore, we still need to focus on the less educated population to prevention and treatment MCC.

Our findings indicated that there are geographic differences in the prevalence of MCC (41.7%, 32.7%, and 25.8%, in northern, southern, and eastern Xinjiang, respectively). The difference was associated with different climatic conditions in these geographic. For instance, using Tianshan as the boundary, northern Xinjiang (Altai and Tacheng areas) has a large temperature difference between winter and summer, with an average annual temperature of -4 to 9℃, annual precipitation of more than 150 to 200 mm, and a frost- free period ranging from 140 to 185 days [34]. In southern Xinjiang (Kashgar and Hotan areas), the annual average temperature is 7 to 14℃, the annual precipitation is 25 to 100 mm, and the frost-free period ranges between 180 and 220 days. On the other hand, east Xinjiang (Hami and Turpan) has an annual average temperature of 13.9℃, while the average highest temperature from June to August is more than 38℃ [35]. Relatively cold geographic are dominated by nomads, and their diets include more alcohol and food with higher levels of animal fat and salt. However, warm and hot areas are mainly farming areas and are inhabited by individuals whose diets include more intake of vegetables and fruits [36]. Recent data from Nanfang et al. indicate that cold climatic conditions are related to increased prevalence of hypertension [37]. Similarly, other studies showed that the MCC rates in adults in southern Brazil and northern Brazil range from 26 to 29% and from 14 to 19%, respectively [38]. In addition, various ethnic groups in Xinjiang live together in groups. Northern Xinjiang is dominated by Kazakhs, Mongolians, and Huis, and most of them are nomads. In contrast, most individuals in southern and eastern Xinjiang are Hans and Uyghurs, who are mainly farmers. This study has also demonstrated that compared with the Han nationality, the Kazakh, Hui, and Mongolian nationalities are at a higher risk for MCC while the Uyghur nationality is at a lower risk for MCC. This data demonstrated that there is a need to formulate geographic -specific and ethnic-specific MCC prevention plans and health services to reduce chronic diseases and MCC for the community-dwelling older adults in Xinjiang.

There were a few limitations associated with this study. Due to the cross-sectional study design, the results of this study can only confirm correlation and cannot infer causal relationships. So, we need to conduct an intervention study such as RCT being a next step to evaluate a program focusing on lifestyle changes and self-management. Nevertheless, our study confirmed for the first time that the prevalence of MCC was different among different gender, age, geographic and ethnic groups in Xinjiang. Older people in northern and southern Xinjiang, and Han, Kazakh, Mongolian and Hui had a higher risk of MCC. In future efforts to prevent chronic diseases, consideration should be given to ethnic and geographical factors, and prevention and treatment measures should be tailored to local conditions and individualized. Early health education, including dietary guidance and medication guidance for chronic diseases, should be provided to improve the health status of the older people, improve their physical functions, and promote healthy aging.

## Conclusion

Among community-dwelling older people in Xinjiang, the prevalence of MCC varies with gender, age, geographic and ethnic group. The risk of MCC is higher in older people living in northern Xinjiang, southern Xinjiang and Han, Kazak, Mongolian and Hui ethnic groups.

## Data Availability

The datasets generated and analyzed during the current study are not publicly available due to this is a newly database which has a lot of important information and we are applying some important projects based on this. For additional information regarding the availability of data contact Dr.Hongmei Wang email: whmdoctor@163.com.

## List of abbreviations

MCC: multiple chronic conditions
HR: heart rate
FBG: fasting blood glucose
Scr: Serum creatinine
BUN: Blood urea nitrogen
TC: serum total cholesterol
TG: triglyceride
LDL-c: low-density lipoprotein cholesterol
HDL-c: high-density lipoprotein cholesterol
ALT: alanine aminotransferase
AST: aspartate aminotransferase
WHO: World Health Organization
OR: Odds ratios
CI: confidence interval
